# The progression of secondary diabetes: A review of modeling studies

**DOI:** 10.3389/fendo.2022.1070979

**Published:** 2022-12-21

**Authors:** Boya Yang, Jiaxu Li, Michael J. Haller, Desmond A. Schatz, Libin Rong

**Affiliations:** ^1^ Department of Mathematics, University of Florida, Gainesville, FL, United States; ^2^ Department of Mathematics, University of Louisville, Louisville, KY, United States; ^3^ Department of Pediatrics, University of Florida, Gainesville, FL, United States

**Keywords:** mathematical model, secondary diabetes, hyperthyroidism, glucocorticoids, epinephrine, growth hormone

## Abstract

Mathematical modeling has provided quantitative information consistent with experimental data, greatly improving our understanding of the progression of type 1 and type 2 diabetes. However, diabetes is a complex metabolic disease and has been found to be involved in crosstalk interactions with diverse endocrine diseases. Mathematical models have also been developed to investigate the quantitative impact of various hormonal disorders on glucose imbalance, advancing the precision treatment for secondary diabetes. Here we review the models established for the study of dysglycemia induced by hormonal disorders, such as excessive glucocorticoids, epinephrine, and growth hormone. To investigate the influence of hyperthyroidism on the glucose regulatory system, we also propose a hyperthyroid-diabetes progression model. Model simulations indicate that timely thyroid treatment can halt the progression of hyperglycemia and prevent beta-cell failure. This highlights the diagnosis of hormonal disorders, together withblood sugar tests, as significant measures for the early diagnosis and treatment of diabetes. The work recapitulates updated biological research on the interactions between the glucose regulatory system and other endocrine axes. Further mathematical modeling of secondary diabetes is desired to promote the quantitative study of the disease and the development of individualized diabetic therapies.

## 1 Introduction

**Figure 1:**
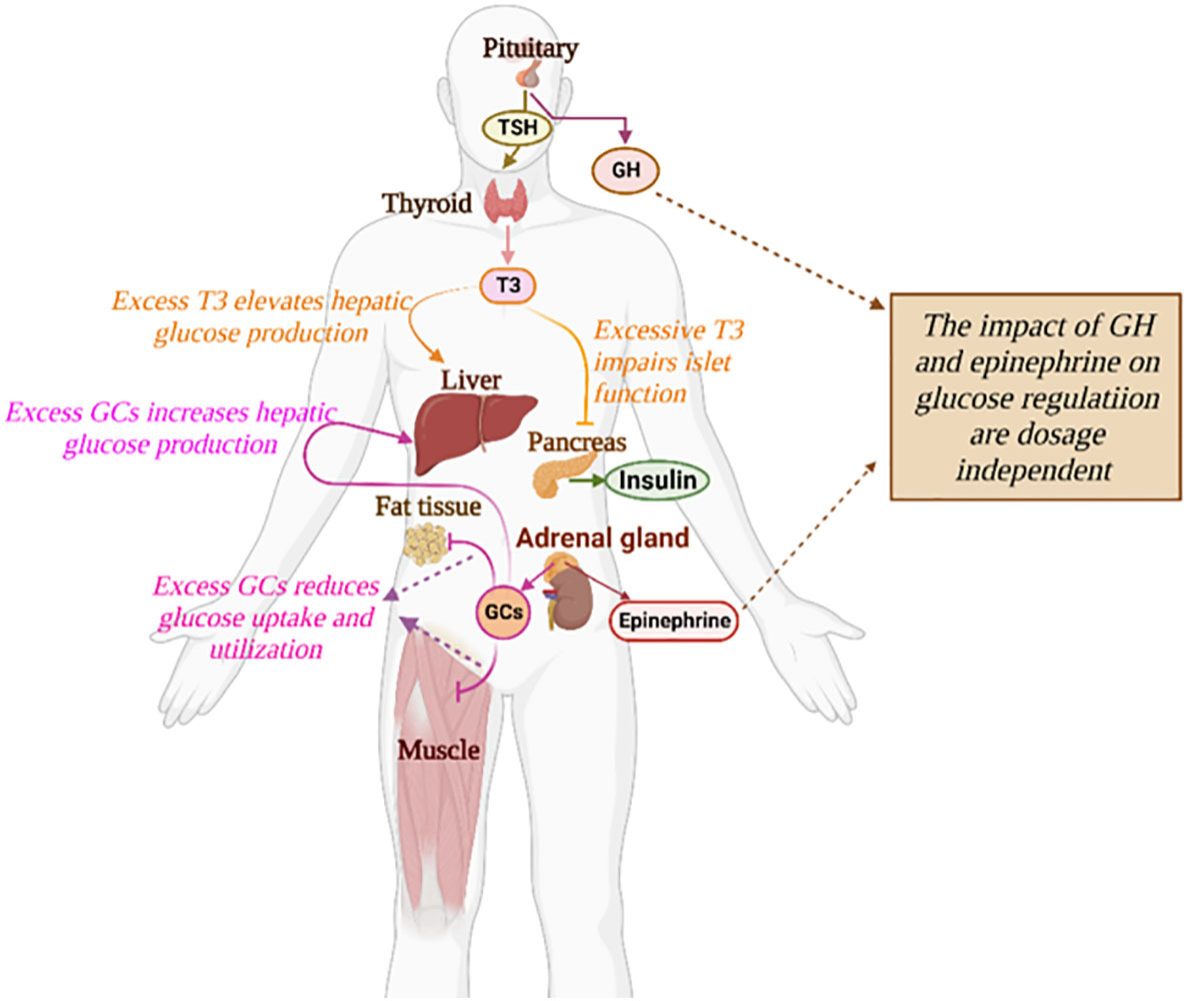
The impact of diverse hormones on the glucose regulatory system. The detailed influences of a specific hormone on glucose regulation are described in text.

Diabetes Mellitus is one of the leading diseases affecting global health and socio-economic development. Diabetes is a condition where the normal glucose-insulin regulatory system is disturbed, the cause of which is multifactorial and complex. Diabetes isclassified into different categories corresponding to distinct pathogeneses, among which, type 1 and type 2 diabetes are the most common (1). Aside from pancreatic hormones, glucose homeostasis is under the control of diverse hormones such as epinephrine, glucocorticoids (GCs), growth hormone, and thyroxine (**Figure 1**). Hormonal diseases have an important impact on glucose control and can exacerbate the progression of diabetes (2). Secondary diabetes is a broad subtype of diabetes including glucose metabolism disorders correlated with various endocrine diseases or medications (1). In recent years, this subgroup has raised the concern of clinicians due to the high incidence of endocrine disorders and drug-induced side effects (3).

Secondary diabetes is commonly involved with acromegaly, hypercortisolism, and thyroid disorders (2, 4). Thyroid disease is the second most frequent endocrine disorder in medical practice following diabetes (5). A number of studies have reported the rising incidence of diabetes mellitus in patients with thyroid hormone dysregulation and vice versa (6, 7). Hypercortisolism is a clinical state attributed to over-exposure to excessive GCs and plays a significant role in the development of diabetes observed in patients subject to chronic stress, Cushing’s syndrome, or long-term GCs treatment (8). Acromegaly is a hormonal disorder caused by the excessive production of growth hormones during adulthood. The prevalence of diabetes in acromegaly varies between 19% to 56% (2). Significantly increased mortality and rate of complications have been reported in these endocrinopathies associated diabetes (2, 9). Compared with patients having type 1 or type 2 diabetes, patients with secondary diabetes are exposed to higher risk and demand intensive treatment. Nevertheless, the management of secondary diabetes is challenging as the patients are already vulnerable to the primary disease. To alleviate the difficulty of managingsecondary diabetes, quantitative approaches to investigate the complex hormone dynamics are desired.

Mathematical models are crucial quantitative tools to test the mechanisms underlying complicated biological systems. Over the past five decades, many mathematical models have been developed to study diabetes facilitating the identification of potential therapies. In particular, mathematical modeling can accelerate the development of the artificial pancreas which provides optimal management of type 1 diabetes (10). The majority of mathematical models are formulated for the study of type 1 and type 2 diabetes (11–14). For example, the type 2 diabetes progression models developed by De Gaetano and his collaborators provide practical approaches in the evaluation of long-term implications of anti-diabetic interventions (15–17). These models were validated by data from the Diabetes Prevention Program study (18, 19), capable of describing the effect of intensive lifestyle intervention and metformin administration, as well as the long-term variation of diagnostic indices in cohorts of virtual patients. Moreover, a physiology-based pharmacokinetic–pharmacodynamic model is proposed by López-Palau et al. to emulate blood glucose dynamics more accurately by including physiological features (20). The work incorporates the effect of gastric emptying and incretin hormones and fits mathematical functions individually to emulate the pathophysiology of type 2 diabetes. However, few models have been developed to investigate secondary diabetes, let alone the models specific to a particular type of secondary diabetes. To promote the mathematical research in secondary diabetes, we will start with a review of major models established for the studies of dysglycemia induced by excessive glucocorticoids, epinephrine, and growth hormone, respectively. These models, which depict the dynamic interactions between hormonal disorders and glucose metabolism, can facilitate the investigation of the underlying mechanisms of secondary diabetes as well as the design of chronomedicine.

To the best of our knowledge, no mathematical models have been developed for the study of progression to secondary diabetes induced by excessive thyroid hormones. We formulate the first hyperthyroid-diabetes model to study the impact of hyperthyroidism onthe progression of diabetes. We investigate the disturbed glucose-insulin dynamics for patients under two different progression rates of hyperthyroidism. The altered glucose-insulin dynamics of hyperthyroid patients after the administration of anti-thyroid drugs are analyzed upon the proposed drug-treatment model. The hyperthyroid-diabetes model enables the quantitative investigation of the hyperthyroid impact on the glucose regulatory system, as well as the delineation of the time course of diabetes remission under anti-thyroid drug treatment, which may assist clinicians in choosing appropriate dosage regimens for patients to achieve euglycemia within a specified time frame.

## 2 Existing models of secondary diabetes

### 2.1 A model of hypercortisolism-induced dysglycemia

Glucocorticoids (GCs) are a class of steroid hormones that have profound effects on energy mobilization, especially glucose metabolism. Synthetic GCs are widely prescribed in medical practice because of their anti-inflammatory, immunosuppressive, and antiallergic effects. However, excess and/or long-term treatment of GCs can induce undesired diabetogenic side effects (21). Aside from drug-induced hypercortisolism, pituitary tumor (Cushing’s disease) and chronic stress are the other two causes of excessive GCs and increase the risk of diabetes development (8). Investigations of the links between glucocorticoid and glucose dynamics are desired to achieve effective glucose control.

Glucocorticoids facilitate the process of gluconeogenesis in the liver, while they reduce glucose uptake and utilization by antagonizing insulin effects in white adipose tissue and skeletal muscle. As a result, over-exposure to GCs leads to hyperglycemia and insulin resistance (22). Although the causal relationship between GCs and dysglycemia is affirmative, the impact of GCs on the pancreatic beta-cells remains debatable (21). Several studies proposed that the effects of synthetic GCs on pancreatic islets and insulin biosynthesis or release depend on the dose and duration of GCs treatment (23, 24). Research in murine models and human studies have shown excess GCs can cause compensatory beta-cell hyperplasia and hyperinsulinemia, with the coexistence of normoglycemia. However, long-term GCs therapy that oversteps the beta-cell compensatory capacity begets impaired insulin secretion, hyperglycemia, and consequent type 2 diabetes (22).

Zavala et al. developed a mathematical model investigating the impact of disrupted cortisol rhythms on the response to oral glucose tolerance tests (OGTT) (25). The model incorporates the effect of transmembrane glucose transporters (GLUTs) on the glucose uptake of fat and skeletal muscle cells, which is under the regulation of both insulin and GCs. In particular, insulin facilitates the translocation of GLUT1, GLUT3 and GLUT4 from intracellular pools to the cell membrane to amplify the glucose uptake in adipocytes and muscle cells, while GCs antagonize this process by translocating GLUTs from the cell membrane back to intracellular compartments (26, 27). In pancreatic beta cells, GLUT1, GLUT2 and GLUT3 are involved in glucose sensing and possess different affinities for extracellular glucose compared to those in fat and muscle cells (26, 28). The model is described by the following ordinary differential equations:


(1) $$\frac{dG\left(t\right)}{dt}=F\left(t\right)+vf_{e}\left(G\right)-a\left[c_{L}f_{L}\left(G\right)+c_{M}f_{M}\left(G\right)\right]T-r_{G}G,$$

(2) $$\frac{dI\left(t\right)}{dt}=\varepsilon +\sigma S_{\beta }\left(G\right)h_{Q}\left(Q\right)-r_{I}I,$$

(3) $$\frac{dT}{dt}=\left(u+v_{I}f_{I}\left(I\right)\right)\left(1-T\right)-\left(d+v_{Q}f_{Q}\left(Q\right)\right)T,$$

Where *G*(*t*) and *I*(*t*) stand for the blood concentrations of glucose and insulin at time *t* (min), respectively; the variable *T*∈(0,1) denotes the fraction of translocatable GLUTs in the cell membrane of peripheral cells, and 1–*T* represents the fraction of GLUTs that docked inside the cell. The functions in Eq. 1-3 are supported by sigmoidal functions with the general form $\phi (x,k_{m},h)=\frac{x^{h}}{x^{h}+k_{m}^{h}}$ , where *k_m_* stands for the half maximum constant and *h* is the Hill coefficient. In the glucose equation, *F*(*t*) represents the glucose boluses from feeding or OGTTs. The term *v_fe_* (*G*) stands for the endogenous glucose production from gluconeogenesis and glycogenolysis, and *v* denotes the maximum rate of the process. The term *at* [*c_L_f_L_* (*G*)+ *c_M_f_M_* (*G*)]*T* represents the glucose uptake by fat and muscle cells, which depends on the fraction of active GLUTs. The factors *c_L_f_L_* (*G*) and *c_M_f_M_* (*G*) stand for the glucose transport mediated by GLUT 1,3 and GLUT 4 respectively, which have different affinities for extracellular glucose. The last term *r_G_G* represents the first-order glucose removal. In theinsulin equation, *ε* stands for the basal insulin secretion rate; *S_β_* (*G*) denotes the glucose sensing in beta-cells; *σ* represents the maximum insulin secretory rate; *h_Q_* (*G*) stands for the regulatory effects of GCs on beta-cell insulin secretion; *r_I_I* represents the first order insulin removal. In the last equation, the term (*u*+*v_I_f_I_* (*I*))(1–*T*) accounts for the translocation rate of GLUTs to the cell membrane, where *u* denotes the basal translocation rate and *f_I_* (*I*) represents the insulin-mediated translocation from the intracellular pools to the cell membrane, at a maximum rate *v*_1_. In the second term, (*d*+ *v_Q_f_Q_* (*Q*))*T* accounts for the translocation rate of GLUTs from the cell membrane down to intracellular pools, where *d* denotes the basal translocation rate and *v_Q_f_Q_* (*Q*) represents the GCs regulated translocation in the same direction.

The model predicts a magnified glucose and insulin non-oscillatory OGTT response under the sub-chronic treatment of dexamethasone (cortisol agonist). In comparison, excess cortisol may enhance the magnitude of the glucose responses to OGTT with maintained circadian and ultradian variability, while greatly suppressing the insulin response and its circadian and ultradian variability. The results also show that excess cortisol results in a right shift of the Starling’s curve toward higher fasting glucose levels, which reveals the impact of hypercortisolism on the progression of diabetes. Overall, this work illustrates how mathematical modeling can provide circadian timing approaches to interpret clinical data, and the potential of mathematical modeling to facilitate the design of the chronotherapies for diabetes secondary to hypercortisolism.

### 2.2 Models studying the impact of epinephrine on glucose regulation

**Figure 2:**
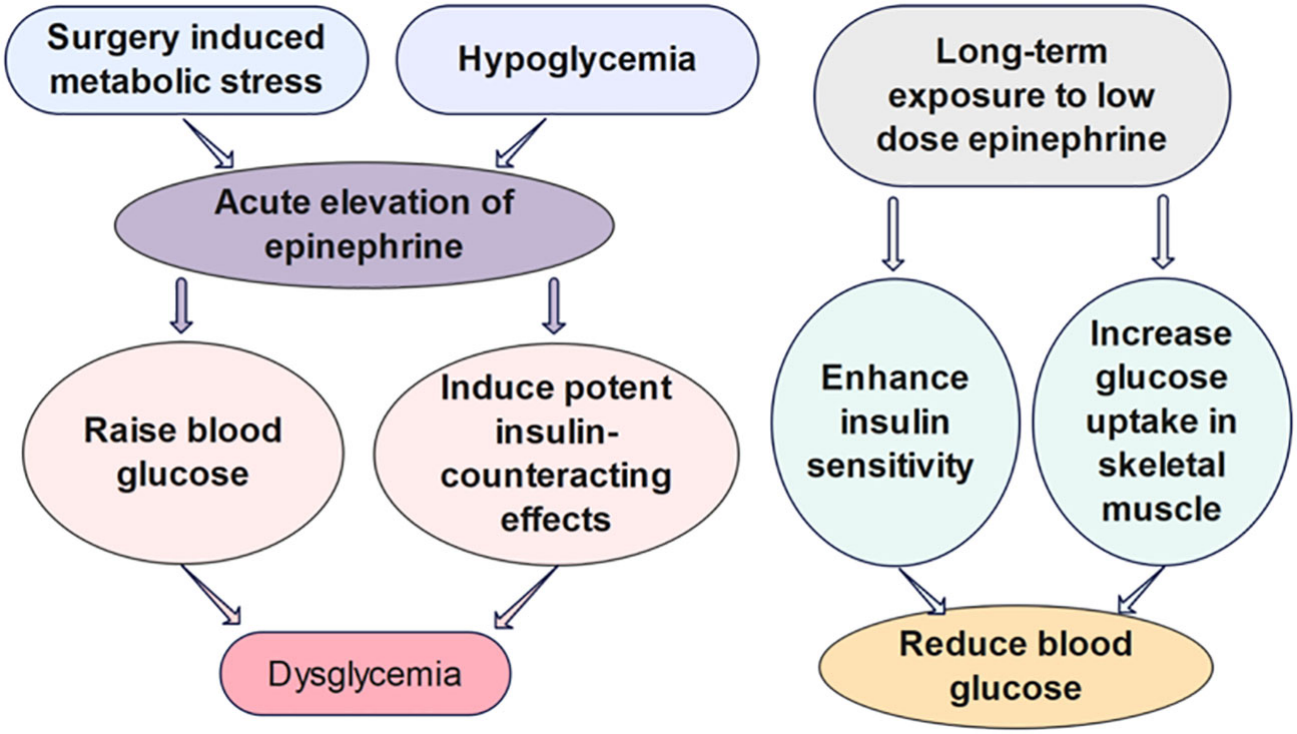
The effects of epinephrine on glucose regulation. Surgery induced metabolic stress, which causes the acute elevation of epinephrine, can significantly increase the rate of dysglycemia of hospitalized patients. The acute influence of epinephrine on the glucose regulation have been shown to be divergent from the impact of chronic infusion of low-dose epinephrine *in vivo* (30–33).

Epinephrine, also known as adrenaline, is a stress hormone that can cause an acceleration in heart rate and glucose metabolism, as well as an increase in blood pressure and muscle strength. The release of epinephrine is typically increased under acute stress to prepare the body for fight-or-flight response (29). As the link between stress and diabetes progression has been brought to the forefront, the impact of epinephrine on glucose metabolism arouses increasing attention from researchers (**Figure 2**). In particular, studies have shown that surgery-induced metabolic stress, which causes the acute elevation of epinephrine, can significantly increase the rate of dysglycemia and the mortality of hospitalized patients (30). In type 1 diabetes, the counterregulatory response of epinephrine to hypoglycemia is an important factor to be considered for the design of glucose control strategies (34, 35).

Several mathematical models have been developed to investigate the quantitative influence of epinephrine on the glucose regulatory system. However, the biological mechanisms some of these models built upon are challenged by further findings of the different metabolic effects of short-term versus long-term epinephrine. Many studies list epinephrine as raising blood glucose, inducing potent insulin-counteracting effects when administered in a short term. Nevertheless, chronic infusion of low-dose epinephrine can enhance insulin sensitivity and glucose uptake in skeletal muscle. The divergent impact may be attributable to the acute versus chronic effects of epinephrine on *β*_2_ stimulation (31). Chronic epinephrine infusion enhanced glycogen synthesis activation and insulin-dependent glucose uptake in rat skeletal muscles (33). Moreover, experiments have shown low dose of *β*_2_-adrenoceptor agonists can improve glucose tolerance in diet-induced obese mice within 4 days of treatment. Prolonged treatment with the low dose of *β*_2_-adrenoceptor agonists can further enhance whole-body insulin sensitivity, immensely reduce hepatic glycogen levels, and lower blood glucose levels (32). Assuming the effects of epinephrine on glucose regulation in animal studies are consistent with human dynamics, a mathematical model should be carefully designed in view of the dosage and duration of epinephrine. We propose that models investigating the impact of epinephrine on the short-term glucose-insulin dynamics should be formulated upon different mechanisms, compared to the models studying the effect on the long-term glucose regulation.

Mohammed et al. established a model with the variables of glucose, insulin, beta-cell mass, and epinephrine to study the glucose regulation under the influence of trauma, excitement and/or stress (36). This model, as shown below, was formulated upon the model of Topp et al. (37), adding the variable of epinephrine to the glucose and insulin equations:


$$\frac{dG}{dt}=R_{0}+G_{e}-\left(E_{GO}+S_{I}I\right)G,$$

$$\frac{dI}{dt}=\frac{\sigma G^{2}}{G^{2}+\alpha }\beta -\left(\rho +k\right)I,$$

$$\frac{d\beta }{dt}=\left(-d+r_{1}G-r_{2}G^{2}\right)\beta ,$$

where *G* (mg/dl), *I* (*μ*U/ml), *β* (mg) represent the blood glucose concentration, insulin concentration, and the mass of functional beta-cells at time *t* (days), respectively. The parameter *R*_0_ stands for the net rate of glucose production per day. The term *S_GO_G* represents insulin-independent uptake of glucose, while *S_I_IG* depicts the insulin-dependent uptake of glucose. In particular, the coefficient *S_I_* (ml/*μ*U/day) represents insulin sensitivity. The insulin secretion from beta-cells is hypothesized to be stimulated by elevated glucose levels in the form of the Hill function, and the parameter *σ* denotes the secretory capacity per beta-cell. The parameter *k* is the insulin clearance rate (/day). The functional beta-cell mass is designed as a second degree polynomial function of glucose with the assumption that moderate glucose level facilitates the growth of beta-cells, while high glucose level aggravates beta-cell apoptosis. The term *G_e_* (mg/dl/day) stands for increasing rate of glucose concentration due to epinephrine, and *ρ* (/day) represents the rate constant of insulin suppression by epinephrine.

As we can deduce from the formulation of the model, Mohammed et al. assumed that epinephrine can raise glucose levels by increasing hepatic glucose production and suppressing insulin secretion. Considering this model was built on Topp’s model, which was developed to study the long-term glucose-insulin dynamics, we expect a better formulation of the model should take the long-term effect of epinephrine into consideration. The enhanced glucose uptake in skeletal muscle and improved insulin sensitivity underthe durable impact of epinephrine can be revealed by adjusting the settings of the model equations. Furthermore, as the long period effect of low-dose epinephrine may reduce the blood glucose level, it seems improper to investigate trauma/stress-induced dysglycemia, focusing only on the impact of epinephrine. For example, exercise, as one of the most potent stimuli to release epinephrine, is a significant approach to ameliorate/prevent diabetes (31). Durable excessive secretion of GCs induced by chronic stress may be a more reasonable factor accounting for the commencement of dysglycemia.

Kwach et al. proposed a model (38) to study the acute influence of epinephrine on short-term glucose-insulin dynamics. The model was built upon the assumption that epinephrine can induce small net stimulation of insulin secretion from pancreatic *β*-cells, which remains debatable as human studies have confirmed the effect of epinephrine on repressing endogenous insulin secretion (39, 40). The assumed self-stimulating effect of epinephrine, presented in the epinephrine equation of the model, needs further justification as well. Kumar and Sandhya filled in biological details of the model of Kwach et al. in their work (41). Their paper cited the study of Sherwin et al. (42), in which the experiment showed that a rapid riseof epinephrine can induce a transient elevation of hepatic glucose output, suppress endogenous insulin secretion, and directly inhibit insulin-stimulated glucose utilization. Nevertheless, the model of Kwach et al. was directly employed in (41) without further modification. The sign of the epinephrine term might be negative in the insulin equation of the model to describe the negative impact of epinephrine on insulin secretion. Examining the biological mechanisms underlying mathematical models isa crucial step to obtaining constructive model implications. A well-developed model for the acute influence of epinephrine on glucose regulation may help to devise a glucose-control strategy for hospitalized patients at risk of hyperglycemia due to surgery-induced metabolic stress.

Type 1 diabetic patients with exogenous insulin therapy are exposed to the risk of hypoglycemia, as their systemic insulin levels may not be reduced in time when the glucose levels begin to decline. In this case, epinephrine becomes the first line of counterregulatory hormone responding to hypoglycemia, due to the early deterioration of glucagon secretion. Moscardó et al. formulated a model to investigate the counterregulatory action of epinephrine during hypoglycemia in type 1 diabetes (43). The model is extended upon the Bergman Minimal Model (44) by adding terms to the glucose equation to account for the influence of epinephrine:


$$\frac{dI}{dt}=\frac{u_{1}\left(t\right)}{Vol_{I}}-nI,$$

$$\frac{dX}{dt}=p_{3}I-p_{2}X,$$

$$\frac{dG}{dt}=p_{4}+\frac{u_{2}\left(t\right)}{Vol_{G}}-p_{1}G-XG+p_{a}A\left(t\right)-p_{h}\max\;(G_{b2}-G,0),$$

$$A\left(t\right)=\{\begin{array}{ll} 0 & \text{if }G\left(t\right)>G_{th}\\ A_{m}\left(t\right)-A_{basal} & \text{if }G\left(t\right)\leq G_{th} \end{array},$$

Where *A*(0) = *A_basal_*, *G*(0) = *G* ^*^. The variables *G*, *I*, and *X* stand for the blood glucose concentration (mg/dl), plasma insulin (*μ*U/ml), and insulin action from a remote compartment (/min), respectively. As the insulin supply for type 1 diabetic patients comes from exogenous infusion, the insulin secretion term is denoted by $\frac{u_{1}\left(t\right)}{Vol_{I}}$ , where *u*_1_(*t*) (*µ*U/min) denotes the insulin infusion rate and *Vol_I_* denotes the insulin distribution volume. The parameter *p*_3_ represents the rate of insulin input in the compartment. The clearance rates of plasma insulin and insulin from a remote compartment are denoted by *n* and *p*_2_ respectively. In the glucose equation, *p*_4_ stands for the hepatic glucose production rate; $\frac{u_{2}\left(t\right)}{Vol_{G}}$ depicts the clamp experimental conditions with the glucose infusion rate *u*_2_(*t*) and the glucose distribution volumes *Vol_G_*; *p*_1_ *G* denotes the glucose uptake independent of insulin and *XG* stands for the glucose uptake rate under the influence of insulin; *A*_m_(*t*) represents the plasma epinephrine concentration (ng/l) and *A_basal_* denotes the basal epinephrine concentration; *G_th_* represents the glucose threshold activating the epinephrine response; thus, *A*(*t*) denotes the epinephrine “effect” which would be activated only after the glucose falls below the glucose threshold; *p_h_max*(*G_b_*_2_–*G*,0) denotes the increase of glucose utilization when glucose stays below a threshold *G_b_*_2_, the value of which is assumed to be in the hypoglycaemic range and greater than *G_th_*. The term *p_h_max*(*G_b_*_2_–*G*,0) is added mainly for a good fit to data.

Studies have shown some diabetic patients under high insulin therapy experienced the stage where the counterregulatory response of epinephrine begins to prevent plasma glucose from further decreasing after the glucose level falls below the activation threshold of epinephrine (around 60 mg/dl) (43). After the epinephrine concentration peaks at the hypoglycaemic plateau, epinephrine secretion rapidly declines when the glucose level starts to recover, returning to its basal level. The work of Moscardó et al. made it possible to present the physiological behavior during hypoglycemia. As this model neglected the inhibitory effect of epinephrine on insulin-dependent glucose utilization in the short term, an improvement over this model may depict a better influence of epinephrine on short-term glucose regulation. Overall, this modeling approach provides a better understanding of the counterregulatory response of epinephrine and may facilitate the design of predictive methods to avoid hypoglycaemic events.

### 2.3 A model investigating the influence of growth hormone on glucose regulation

Growth hormone (GH) or somatotropin, is an important peptide hormone that stimulates growth, cell reproduction, and regeneration. GH also stimulates the production of insulin-like growth factor 1 (IGF-1), a hormone similar to insulin in molecular structure (45). GH has been characterized as one of the anti-insulin hormones. The diabetogenic effect of GH is also validated by the high prevalence of diabetes in patients with acromegaly, a condition wherein the excessive growth hormones are produced citep (29). The risk of hyperglycemia exists likewise in patients with GH deficiency who need the GH administration for treatment. Large-scale cohort studies have shown that, compared to the general population, the incidence of developing type 2 diabetes for children under GH treatment was increased more than six times, especially in patients with predisposing risk factors, such as obesity (46–48).

**Figure 3:**
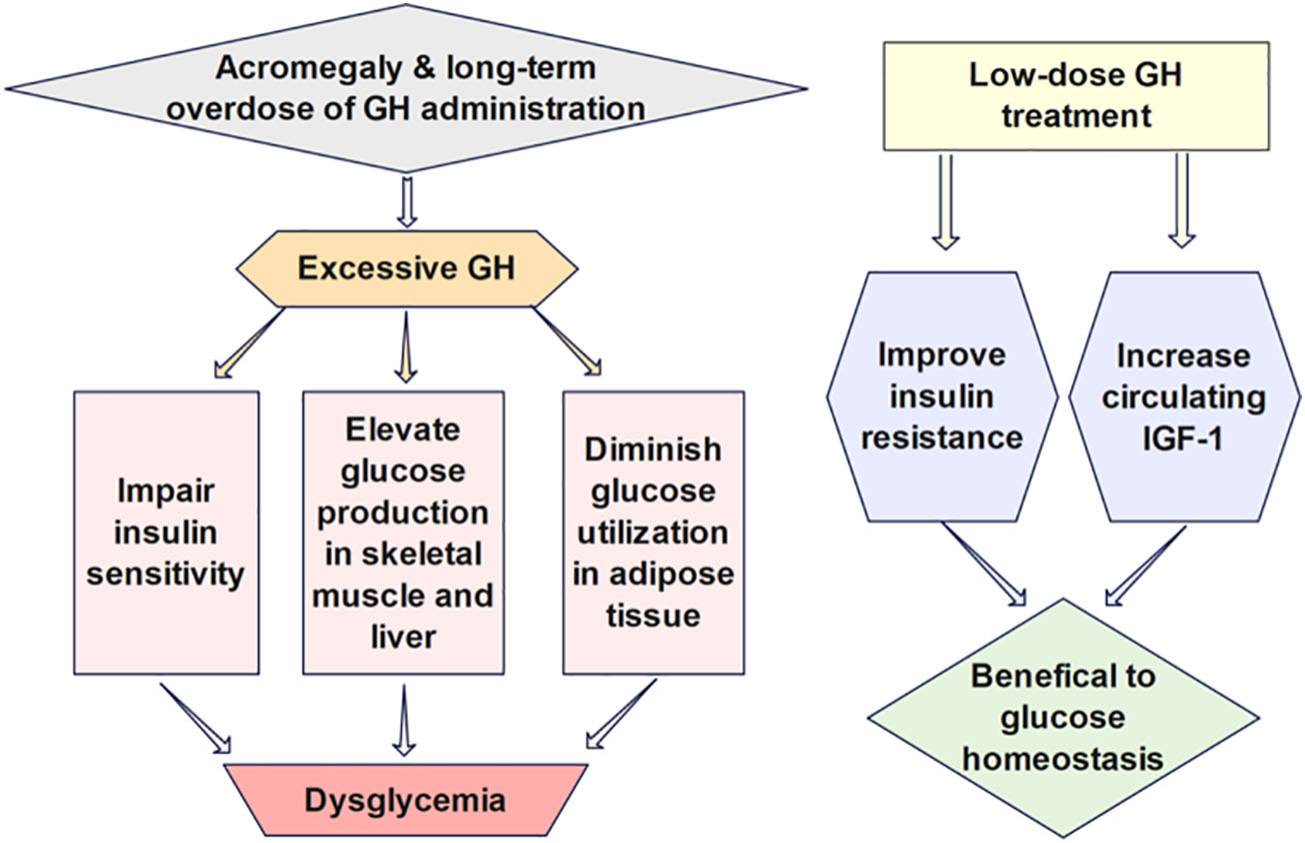
The effects of GH on glucose regulation are intricate partially due to its indirect impacts *via* IGF-1, which has glucose-lowering functions analogous to insulin. Low-dose GH has been shown to benefit insulin sensitivity and glucose homeostasis due to increased circulating IGF-1, while long-term GH treatment in high doses impairs insulin sensitivity and exacerbates insulin resistance.

The effects of GH on glucose regulation (**Figure 3**) are intricate partially due to its indirect impacts *via* IGF-1, which has glucose-lowering functions analogous to insulin (49). GH can elevate glucose production in the skeletal muscle and liver and diminish glucose utilization in adipose tissue by antagonizing the action of insulin. Insulin secretion is also enhanced for the compensation of elevated blood glucose after GH administration (50). Prior studies have demonstrated that low-dose GH may have beneficial effects on insulin resistance and glucose homeostasis due to increased circulating IGF-1, while long-term GH treatment in high doses impairs insulin sensitivity and exacerbates insulin resistance (51). Thus, cautious monitoring of the possible adverse impact on glucose metabolism induced by GH treatment is advocated. Mathematical models investigating the quantitative influence of growth hormone on the long-term glucose dynamics may facilitate the examination of the durable effect of GH therapy.

Alali et al. developed a model studying the effect of growth hormone on glucose homeostasis (52). The model, as shown below, is extended upon the model of Boutayeb et al. (53), including an equation of GH to depict the interaction of GH withglucose and free fatty acids (FFA):


$$\frac{dG}{dt}=a-\left(b+cRI\right)G+m_{1}\left(F-F_{b}\right)+cGH,$$

$$\frac{dI}{dt}=\frac{\beta dG^{2}}{\left(e+G^{2}\right)\left(1+R\right)}-fI-fRI,$$

$$\frac{d\beta }{dt}=\left(-g+hG-iG^{2}\right)\beta ,$$

$$\frac{dR}{dt}=j\left(1-R\right)-kIR-lR,$$

$$\frac{dF}{dt}=-m_{2}\left(F-F_{b}\right)+m_{3}\left(G-G_{b}\right)+x\left(GH-GH_{b}\right),$$

$$\frac{dGH}{dt}=p-\omega GH-s\left(F-F_{b}\right)-zR,$$

Where *G*, *I*, *β*, *R*, and *F* stand for the blood glucose level (g/l), plasma insulin concentration (*μ*U/ml), *β*-cell mass (mg), the fraction of insulin receptors on the membrane of the muscle cells, and the concentration of FFA (*μ*mol/l) at time *t* (days), respectively. The parameter *a* denotes the constant secretion rate of glucose by the liver and kidneys. The term *b* + *cRI* denotes the total body glucose utilization rate. The FFA-induced glucose production is represented by *m*_1_(*F*–*F_b_*), and the GH-induced glucose production through gluconeogenesis and glycogenolysis is denoted by *cGH*. In the insulin equation, the factor $\frac{G^{2}}{\left(e+G^{2}\right)}$ depicts the sigmoidal relationship between the extracellular glucose concentration and the insulin secretion; $\frac{d}{1+R}$ represents the insulin secretion factor per *β* cell; *fI* stands for the insulin clearance by liver and kidneys; *fRI* denotes the insulin clearance by the muscle cell receptors. The *β*-cell equation follows the same formulation in Topp’s model (37). In the receptor equation, *j* represents the recycling rate of internalized receptors: *k* stands for the insulin-induced down-regulation rate of receptors on the cell membrane; *l* denotes the clearance rate of the surface receptors. The parameters *F_b_*, *G_b_*, and *GH_b_* represent the basal concentration of FFA, glucose, and growth hormones, respectively. The term *m*_3_(*G*–*G_b_*) denotes the lipogenesis rate induced by excess glucose, and *x*(*GH*–*GH_b_*) represents the lipolysis rate stimulated by GH. The clearance rate of FFA is denoted by *m*_2_(*F*–*F_b_*). In the GH equation, *p* represents the production rate of GH by the somatotropic cells; *ωGH* stands for the clearance of GH by the liver; the GH uptake by fat cells and receptors is represented by *s*(*F*–*F_b_*) and *zR*, respectively.

This work provides the first mathematical model that incorporates GH into the glucose regulatory system. The receptor equation obtained from the base model in (53) impedes this model from data fitting and applications. In addition, the insulin equation cited from the base model may be questionable. The variable *R* was designed to denote the fraction of insulin receptors on the membrane of the muscle cells, while the term $\frac{d}{1+R}$ was claimed to represent the insulin secretion factor per *eta* -cell. The impact of the changed behavior of insulin receptor can be implied by designing the insulin sensitivity coefficient *c* to vary with other variables in the system. Moreover, as *β*-cell function and insulin level play a significant role in the glucose regulation, incorporating the variable GH into the insulin and *β*-cell equation is important for a better investigation of the influence of GH on glucose dynamics. Further modeling work studying the interaction between GH and glucose regulation is desired to help unravel the intricate physiological effect of GH with different dosages and treatment duration.

## 3 Hyperthyroidism-induced diabetes

**Figure 4:**
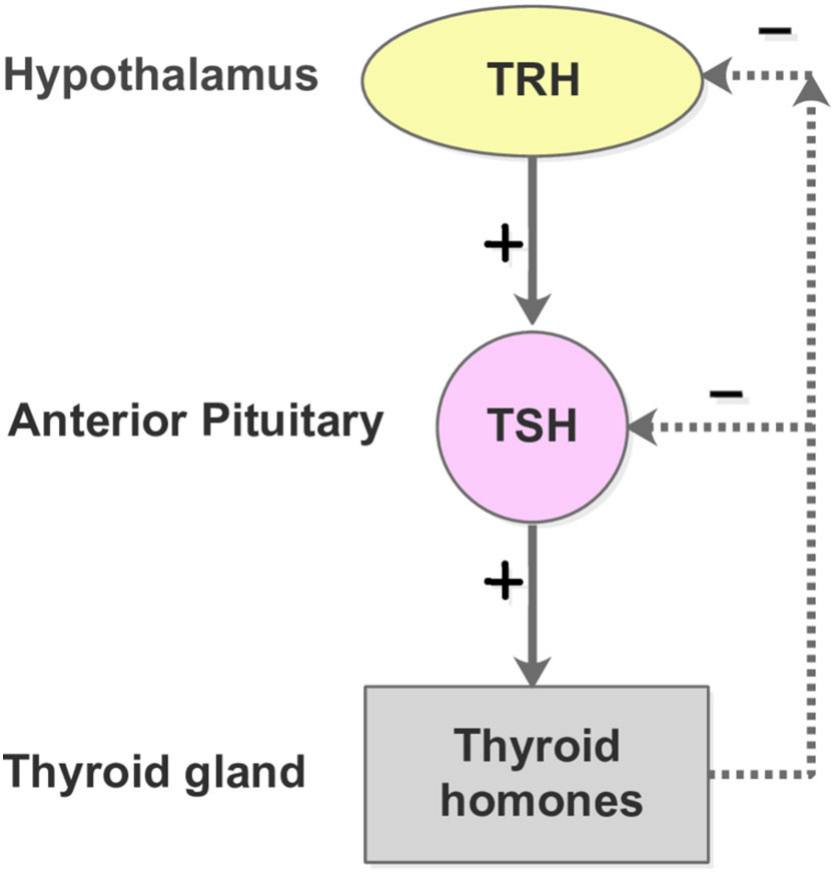
The HPT-axis negative feedback mechanism. The free T3 and T4 concentrations lower than their respective normal set point values lead to the secretion of TRH from the hypothalamus. The pituitary gland is subsequently promoted to produce and secrete TSH into the blood, which in turn stimulates the thyroid follicle cells to secrete T3 and T4. In contrast, when the plasma levels of free T3 and T4 are elevated beyond their normal range, the hypothalamus and pitutary gland respond by reducing the secretion of TRH and TSH, which slows down the production of T3 and T4.

The thyroid is an endocrine gland in the neck that secretes triiodothyronine (T3) and thyroxine (T4) (54). Both high and low levels of thyroid hormones can produce adverse effects on the body. The production of T3 and T4 is under the control of thyroid-stimulating hormone (TSH) and thyrotropin-releasing hormone (TRH). This secretory system is sequential: the hypothalamus secretes TRH, stimulating the anterior pituitary gland to produce TSH, after which, the TSH stimulates the thyroid to generate T3 and T4. If the thyroid hormones become overly elevated in the blood, the TSH levels are suppressed in response, lowering the thyroid hormone secretion (55). The negative feedback control of the Hypothalamus-Pituitary-Thyroid (HPT) axis, as shown in **Figure 4**, maintains the thyroid hormone regulation, forming the set points of T3, T4, and TSH.

An elevated risk of T2DM in patients with hyperthyroidism has been documented (6, 7). We propose a hyperthyroidism-induced diabetes model based on our previous work of a generalized diabetes progression model (56):


$$\frac{dG}{dt}=G_{in}+p_{1}\left(X\right)-f_{2}\left(G\right)-C\left(I\right)GI,$$

$$\frac{dI}{dt}=f_{1}\left(G\right)p_{2}\left(X\right)\beta -kI,$$

$$\frac{d\beta }{dt}=\left(f_{3}\left(I\right)+p_{3}\left(X\right)\right)\beta ,$$

where *f*_2_(*G*) = *g*_1_ *G*, $C\left(I\right)=r_{0}+\frac{r_{1}}{r_{2}+e^{r_{3}I}}$ , $f_{1}\left(G\right)=\frac{s_{1}G^{2}}{G^{2}+s_{2}}$ , $f_{3}\left(I\right)=\frac{m_{1}I}{I^{2}+{\left(m_{2}\right)}^{2}}-m_{3}$ . Here, *G* (mg/dl), *I* (*μ*U/ml), *β* (mg) represents the plasma glucose concentration, insulin concentration, and the mass of functional beta-cells (preserving appropriate insulin production and secretion) at time *t* (days), respectively. The parameter *G_in_* depicts the sum of the average glucose uptake rate and the hepatic glucose production per day. The term *f*_2_(*G*) represents the insulin-independent uptake of glucose, while *C*(*I*)*GI* stands for the insulin-dependent uptake of glucose. The term *f*_1_(*G*) represents the beta-cell secretory function (the ability to produce, store and release insulin)per cell, and *k* (/day) denotes the insulin clearance rate. The function *f*_3_(*I*) represents the net growth rate of functional beta-cell mass that depends on the insulin level. Moreover, we incorporate an interference factor *X* into the glucose regulatory model, accounting for the progressive impact of the environmentally induced or epigenetic-related diabetogenic factor on the glucose regulation. The function *p*_1_(*X*) stands for the increased hepatic glucose production caused by the pathological factor; *p*_2_(*X*) represents the impact of the factor on the insulin secretion rate; *p*_3_(*X*) describes the abnormal response of beta-cells to a hostile environment that develops in a slow time scale. All parameters in the model are positive.

The underlying mechanism of the impact of hyperthyroidism on the deterioration of glucose control has been widely investigated in the literature (57). As a larger deviation of the T3 level from its set point (denoted by the parameter *U*) leads toa worse impact on the *GIβ* regulatory system, we quantify the hyperthyroid factor *X* by $\frac{\left|T_{3}-U\right|}{U}$ and integrate its impact to the glucose regulatory system with the influence functions *p_i_* (*X*) (*i* = 1,2,3). Prior research has shown that excess thyroid hormones can increase hepatic glucose production through the elevated hepatic expression of glucose transporters as well as enhanced glycogenolysis and gluconeogenesis activities (58). We design the elevated hepatic glucose production *p*_1_(*X*) to be a power function of *X*, which can be determined by the extent to which the hyperthyroid factor impacts the glucose generation rate. Moreover, T3 exerts profound effects on the proliferation of pancreatic islet cells and insulin secretion (57, 59). Increased secretion of insulin and elevated fasting insulin are observed in hyperthyroidism (60, 61). We thus assume the beta-cell secretion function is linearly increasing with *X*, as shown in Eq. 5. Furthermore, excess T3 leads to considerable impairment of the islet function, while physiological T3 treatment promotes beta-cell proliferation (57, 62). In view of this, we assume functional beta-cell mass undergoes the influence of *X* in a pattern of a downward parabola and formulate the function *p*_3_(*X*) with the form in Eq. 6.


(4) $$p_{1}\left(X\right)=h_{1}X^{\alpha },$$

(5) $$p_{2}\left(X\right)=1+h_{2}X,$$

(6) $$p_{3}\left(X\right)=q_{1}X\left(q_{2}-X\right).$$

Although T3 is the biologically active thyroid hormone in target tissues, approximately 80% of the T3 production in humans comes from the deiodination of T4 (63). The deiodination activity in human involves two iodothyronine deiodinases (D2 and D3), the interactionsbetween which are complex and vary with different physiological conditions (64–66). The majority of T3 and T4 are bound to thyroglobulin in blood, and the fraction that can flow freely in the blood are abbreviated as FT3 and FT4. The lab testof FT4 is considered as a more accurate evaluation of thyroid hormone concentration than the measurement of total T3, T4 and FT3 due to the limitation of measuring technique (67). In addition, serum TSH concentration is regarded as a more robust index of the thyroid hormone status. Therefore, physicians generally prescribe patients the blood test of FT4 and TSH to assess their thyroid condition (68). To formulate a simple and attractive model that only involves the essential components of the complex endocrine subsystems and that is accessible to data fitting, we assume the elevated blood FT4 concentration can indicate the excessive intracellular T3 level in hyperthyroidism and replace the variable T3 with FT4 in the model. The dynamics of FT4 are investigated based upon our previous study of thyroid hormone regulatory system (55). We note the amount of FT4 is always greater than its euthyroid set points U during the progression from euthyroidism to hyperthyroidism. Therefore, we construct the following system to study the impact of hyperthyroidism on the diabetes progression.

### 3.1 Hyperthyroid-diabetes progression model

The hyperthyroid-diabetes model without hyperthyroid treatment is given by


$$\frac{dG}{dt}=G_{in}+p_{1}\left(\frac{FT4-U}{U}\right)-f_{2}\left(G\right)-C\left(I\right)GI,$$

$$\frac{dI}{dt}=f_{1}\left(G\right)p_{2}\left(\frac{FT4-U}{U}\right)\beta -kI,$$

$$\frac{d\beta }{dt}=\left[f_{3}\left(I\right)+p_{3}\left(\frac{FT4-U}{U}\right)\right]\beta ,$$

$$\frac{dFT4}{dt}=\frac{a_{1}\left(t\right)TSH}{b_{1}+TSH}-d_{1}\text{FT}4,$$

$$\frac{dTSH}{dt}=a_{2}-\frac{a_{2}\left(FT4-U\right)}{b_{2}+FT4}-d_{2}\text{TSH},$$

where *a*_1_(*t*) stands for the time-dependent FT4 synthesis factor; *b*_1_ represents the TSH concentration corresponding to half the maximal synthesis rate of FT4; *d*_1_ is the decay rate of FT4; *a*_2_ represents the default release rate of TSH from the pituitary when FT4 reaches the euthyroid set point value; *b*_2_+2*U* is the concentration of FT4 resulting in half the maximal inhibitory effect on the TSH secretion rate controlled by the pituitary; *d*_2_ represents the decay rate of TSH. The expression of *a*_1_(*t*) varies among individuals with different progression rates of hyperthyroidism.

By setting the coefficients of *X* in *p_i_* (*X*) to be close to zero, our hyperthyroid diabetes model can illustrate the case where diabetic patients are free from hyperthyroidism. In the clinical scenario, some patients with hyperthyroidism develop diabetes over time, while others can stay away from diabetes in life time. The diverse genetic traits of individuals may determine their cellular response to the hyperthyroid factor, which can be expressed by different parameter values in *p_i_* (*X*).

### 3.2 Hyperthyroid-diabetes model under hyperthyroid treatment

It has long been recognized that the treatment of hyperthyroidism can improve glucose control. A nation-wide cohort study shows that the treatment of thyroid dysfunctions can reduce the manifestation of T2DM (69). To quantitatively analyze the benefits of hyperthyroid treatment to the glucose regulatory system, we incorporate the drug treatment to the FT4 equation and formulate the hyperthyroid-diabetes model under treatment as follows:


$$\frac{dG}{dt}=G_{in}+p_{1}\left(\frac{FT4-U}{U}\right)-f_{2}\left(G\right)-C\left(I\right)GI,$$

$$\frac{dI}{dt}=f_{1}\left(G\right)p_{2}\left(\frac{FT4-U}{U}\right)\beta -kI,$$

$$\frac{d\beta }{dt}=\left[f_{3}\left(I\right)+p_{3}\left(\frac{FT4-U}{U}\right)\right]\beta ,$$

$$\frac{dFT4}{dt}=\frac{a_{1}\left(t\right)TSH}{b_{1}+TSH}-d_{1}\text{FT}4-\frac{d_{3}D}{D+IC_{50}}FT4,$$

$$\frac{dTSH}{dt}=a_{2}-\frac{a_{2}\left(FT4-U\right)}{b_{2}+FT4}-d_{2}\text{TSH},$$

where *D* represents the anti-thyroid drug (ATD, e.g. Carbimazole) dosage (mg); *D*_3_ stands for the maximum reduction rate of FT4 caused by drug intake; *IC*_50_ represents the dosage of the ATD that achieves half of the maximum reduction rate (70).

### 3.3 Influence of hyperthyroidism on the progression of diabetes

Because patients have varied rates of hyperthyroid progression, we investigate the *GIβ* dynamics for virtual patients under two different progression rates of hyperthyroidism. We adopt (7-18 pg/ml) and (0.4-4 mU/l) as the normal reference range for the FT4 and TSH respectively (71, 72), and assume U=12.5 pg/mL (the average value of the lower and upper bound of FT4) as the set point of FT4 for the patients we study below. Notably, an apparent deviation from the set point value of FT4 without crossing the bounds of the inter-individual reference range, is sufficient to cause physiological impact on the patients. The American Diabetes Association (ADA) characterizes the fasting glucose levels of euglycemia, pre-diabetes, and diabetes as less than 100 mg/dl, 100-125 mg/dl, and greater than 125 mg/dl, respectively (73). These ranges are contingent on the source and may vary slightly across different labs. In our work, we consider 5 - 20 *μ*U/mL as the reference range for normal fasting insulin and *I*≥25*μ* U/mL as the criterion of hyperinsulinemmia (74–76). The model parameters are listed in **Table 1**, where the values of *h*_2_, *b*_1_, *a*_2_, *b*_2_ are chosen to ensure the consistency between simulated hormone dynamics and clinical observations. The remaining parameters are adopted from our previous work (56) where the model was validated by the Pima Inidan data.

**Table 1:** Parameter values for the hyperthyriod-diabetes progression model.

Parameters	Value		Source
*r*_0_	0.019	*ml*·*μU* ^–1^·*day* ^–1^	(56)
*r*_1_	1.98	*ml*·*μU* ^–1^·*day* ^–1^	(56)
*r*_2_	3.088	—	(56)
*r*_3_	0.05	—	(56)
*G_in_*	864	*mg*·*dl* ^–1^·*day* ^–1^	(37)
*g*_1_	1.44	*day* ^–1^	(37)
*s*_1_	86.4	*μU*·*ml* ^–1^·*day* ^–1^	(56)
*s*_2_	20000	*mg* ^2^·*dl* ^–2^	(37)
*k*	432	*day* ^–1^	(37)
*m*_1_	0.1	*day* ^–1^	(56)
*m*_2_	100	*μU*·*ml* ^–1^	(56)
*m*_3_	0.004	*day* ^–1^	(56)
*h*_1_	300	*mg*·*dl* ^–1^·*day* ^–1^	(56)
*α*	$\frac{1}{3}$	—	(56)
*h*_2_	1	—	see text
*q*_1_	0.04	*day* ^–1^	(56)
*q*_2_	0.5	—	(56)
*n*_1_	0.0005	—	(56)
*n*_2_	1	—	(56)
*b*_1_	2.75	*mU*·*L* ^–1^	see text
*d*_1_	0.099	*day* ^–1^	(55)
*U*	12.5	*mmol*·*L* ^–1^	see text
*d*_2_	16.6355	*day* ^–1^	(55)
*a*_2_	10	*mU*·*L* ^–1^·*day* ^–1^	see text
*b*_2_	1	*mmol*·*L* ^–1^	see text

**Figure 5:**
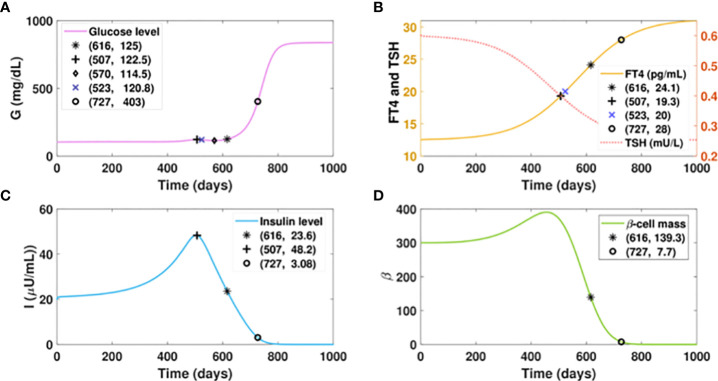
Dynamics of glucose, insulin, and functional beta-cell mass level with fast progressing hyperthyroidism. **(A-D)** Time evolution of blood glucose concentration, plasma FT4 and TSH concentrations, insulin level, and functional betacellmass. The initial conditions of FT4 and TSH are set to be 12.5 pg/ml and 0.6 mU/l, respectively. The parametervalues are listed in **Table 1**. We assume the FT4 synthesis factor for patient A to be $a_{1}\left(t\right)=6.9+\frac{30}{1+e^{\left(6-0.01t\right)}}$ . Withthe gradual elevation of FT4, the glucose level of this patient would reach 122.5 mg/dl on day 507. The glucose wouldbe dragged down slightly to 114.5 mg/dl in 63 days by improved insulin sensitivity. However, the glucose level wouldrise again thereafter subsequent to the continuous decline of insulin, which is caused by the damage of excessive thyroidhormones to beta-cells. With the gradual elevation of FT4 for 1.7 years, the glucose level of patient A would cross 125mg/dl, the threshold of overt diabetes. As the FT4 level further increases, a complete beta-cell failure occurs and drivespatient A to the late stage of diabetes.

We first consider a virtual patient A who is euglycemic and would develop hyperthyroidism with a fast progression rate, as shown in **Figure 5B**. **Figure 5A** shows that the increasing amount of excess thyroid hormones drives the gradual and continuous elevation of the glucose level (fasting) before FT4 reaches 19.3 pg/ml. Although the glucose level would be dragged down by 8 mg/dl in 63 days by improved insulin sensitivity, the rising excessive thyroid hormones start deteriorating beta-cells afterwards. The continuous decline of insulin and persistent escalation of glucose follow subsequently. After the gradual elevation of FT4 for 1.7 years, the glucose level of this virtual patient would reach 125mg/dl, the threshold of the diabetic stage. When the FT4 level exceeds its set point value by 124%, a complete beta-cell failure occurs and drives patient A to the late stage of diabetes. The observation of fluctuated blood glucose level is common in clinical settings (77).

**Figure 6:**
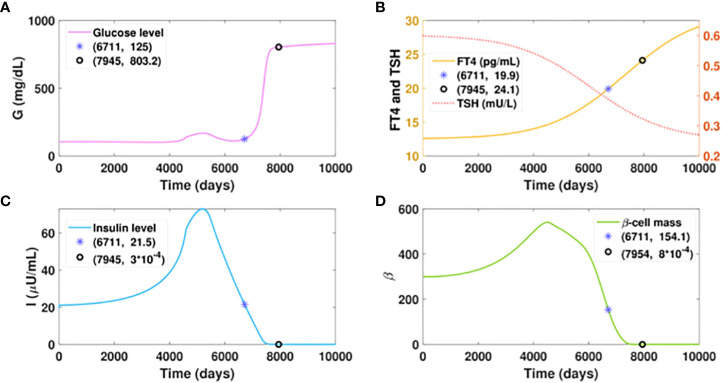
Dynamics of glucose, insulin, and functional beta-cell mass level with slowly progressing hyperthyroidism.Here $a_{1}\left(t\right)=6.9+\frac{30}{1+e^{\left(6-t/1300\right)}}$ . All the other parameter values and initial conditions remain the same as in **Figure 5**. **(A-D)** Time evolution of blood glucose concentration, plasma FT4 and TSH concentrations, insulin level, and functionalbeta-cell mass. Graph (b) shows patient B would reach the FT4 level of 20 mg/dl in approximately 18.5 years, whichis delayed 17 years than patient A. The time for him to develop diabetes would be postponed for 16.7 years, comparedwith patient A. However, patient B would step into the late stage of diabetes with an FT4 level of 24.1 pg/ml, whilepatient A just cross the threshold of diabetes with the same level of FT4.

Suppose virtual patient B develops hyperthyroidism at a slow rate, spending an extra 17 years more than patient A to reach the FT4 level of 20 pg/ml, as presented in **Figure 6B**. The time for this patient to develop diabetes would be postponed for 16.7 years compared to patient A, which exhibits the benefit of delayed hyperthyroid progression on slowing the course of diabetes. However, compared to the glucose level of 125 mg/dl that patient A would develop with the FT4 of 24.1 pg/ml, patient B would be in the late stage of diabetes with the same FT4 level. Additionally, the elevated glucose level of patient B is overwhelmingly higher than that of patient A, when their FT4 levels both increase from 20 pg/ml to 24 pg/ml. These results indicate that aside from the amount of excess thyroid hormones, the duration of hyperthyroid exposure affects the severity of hyperglycemia.

### 3.4 Significance of hyperthyroid treatment for glucose control

**Figure 7:**
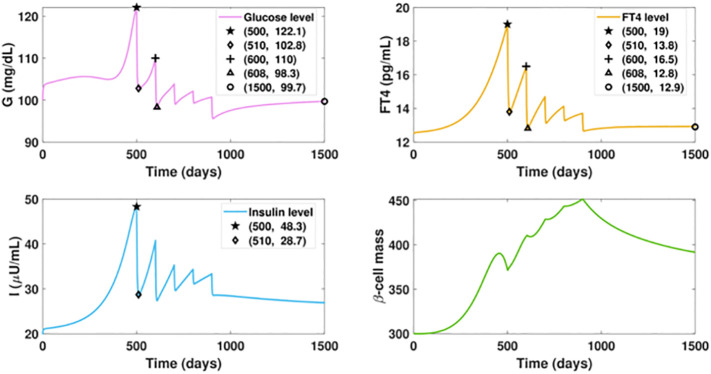
Dynamics of altered glucose, insulin, and functional beta-cell mass levels with timely treated hyperthyroidism. The ATD dosage *D* is designed as a piecewise function in the following pattern: *D* = 0, for *t* ≤ 500; *D* = 3, for 500< *t* ≤ 600; *D* = 8, for 600< *t* ≤ 700; *D* = 12, for 700< *t* ≤ 800; *D* = 15, for 800< *t* ≤ 900; *D* = 20, for *t* > 900. The parameter values in the drug intervention term are: *d*_3_ = 1, *IC*_50_ = 30. All the other parameter values and initial conditions remain the same as those in **Figure 5**. The initial rise of FT4 level would cause a temporary upsurge of glucose level around the initial point, which is then quickly counterbalanced by the fast increase of insulin levels. In contrast with the increasing trend of the glucose level in Figure fFT4fastProgressingHyper(a), the administration of 3mg ATD drug after day 500 can drag the FT4 level down by 5.2 pg/ml in 10 days, followed by the 19.3 units reduction of glucose level and 19.6 units decrease of insulin level. However, the dose is not enough to prevent the FT4 level from increasing after day 510, which would result in the rise of glucose. We then increase the ATD dose to 5 mg after day 600. As a result, the elevated dosage would drive the FT4 down close to its set point value, and the glucose level would decrease again. Yet this level of treatment is insufficient as the inherent deterioration of the thyroid remains. To enhance the treatment, an increased dosage is applied every one hundred days until day 900 when the dosage becomes fixed. At the end, the FT4 level approaches the steady state with 12.9 pg/ml and the glucose level would be regulated within the normal range after day 1500.

We investigate the influence of drug treatment on the *GIβ* dynamics for the fast progression of hyperthyroidism. The results of timely treated hyperthyroidism are shown in **Figure 7**. We assume the ATD drug is administered daily after day 500 with the dose increasing intermittently, that is, the dosage *D* is designed as a piecewise function, the specific expression of which is described in the caption of **Figure 7**. The graphs depict that the initial rise of FT4 level would cause a temporary upsurge of glucose level around the initial point of glucose, which is then quickly counterbalanced by the fast increase of insulin levels. In contrast with the increasing trend of the glucose level in **Figure 5A**, the administration of 3mg ATD drug after day 500 would result in a sharp reduction of both the glucose level and the insulin level in 10 days.

However, the dose is not enough to counteract the impact of the hyperthyroid factor, and the FT4 level would be elevated again, leading to an upsurge of the glucose level. We then increase the ATD dose to 5 mg for the next 100 days. As a result, the elevated dosage would drive the FT4 down close to its set point value. Subsequently, the glucose level decreases again. Yet this level of treatment is insufficient as the inherent deterioration of the thyroid remains, a condition demonstrated in **Figure 5B**. To enhance the treatment, an increased dosage is applied every one hundred days until day 800 when the dosage becomes fixed. Consequently, the FT4 level approaches the steady state with 12.9 pg/ml, an improvement over the steady state of **Figure 6B**. Moreover, the glucose level would be regulated within the normal range after day 1500, a dramatic improvement relative to **Figure 5A**. In contrast with the massive mortality of beta-cell and the deficient insulin level exhibited in the late period of **Figure 5**, the thyroid treatment prevents beta-cell failure and the occurrence of insufficient insulin. Overall, the result highlights that the management of thyroid dysfunction may be of primary consideration for the therapy of diabetic patients.

**Figure 8:**
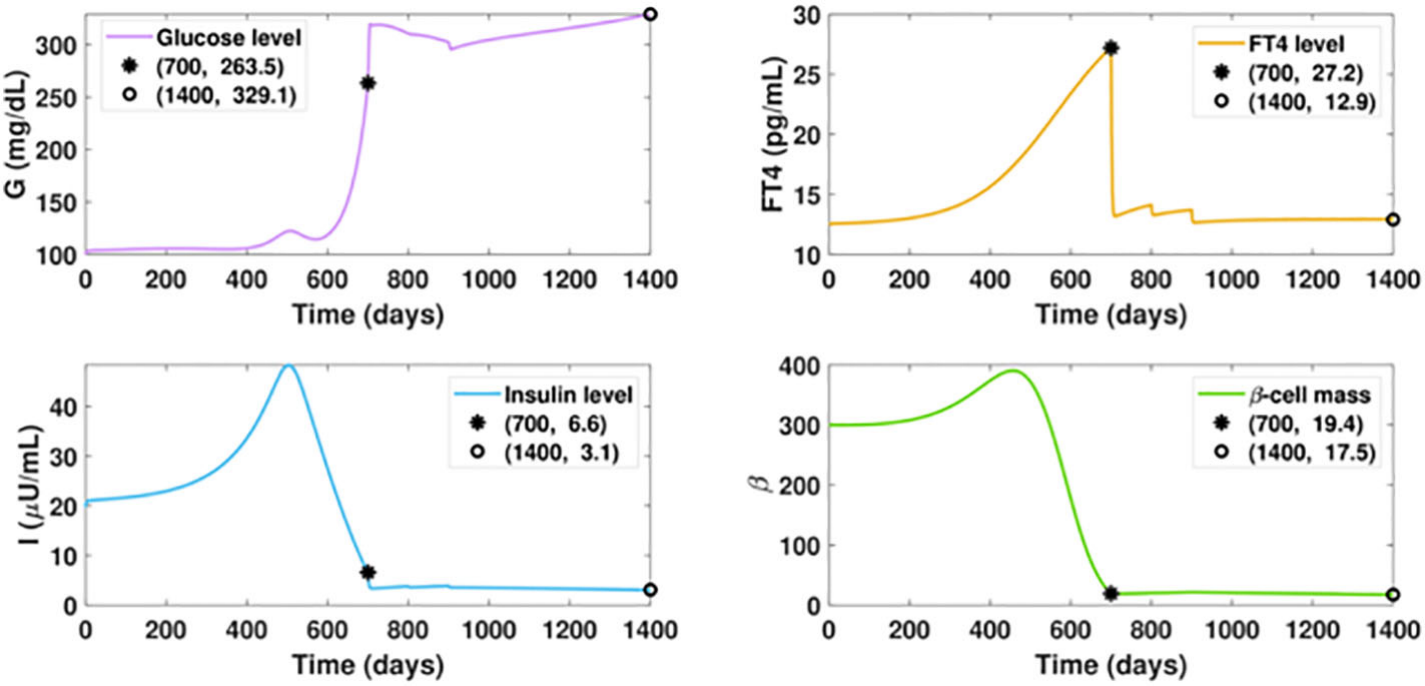
Dynamics of altered glucose, insulin, and functional beta-cell mass levels with belatedly treated hyperthyroidism. The ATD dosage *D* is designed as a piecewise function in the following pattern: *D* = 0, for *t* ≤ 700; *D* = 12, for 700< *t* ≤ 800; *D* = 15, for 800< *t* ≤ 900; *D* = 20, for *t* > 900. All the other parameter values and initial conditions remain the same as those in **Figure 7**. The anti-hyperthyroid treatment starts at day 700, when the beta-cell defect already occurred. Although the FT4 level sharply decreases and approaches the set point value after day 1000, there is no apparent mitigation of diabetes, which is in significant contrast with the outcome of timely treated hyperthyroidism.

Notably, our simulation results indicate belatedly treatment of hyperthyroidism fails to reverse diabetes. In **Figure 8**, we assume the ATD drug is initiated on day 700 when the FT4 level is 8.2 pg/ml higher than the value on day 500. The dosage regimen in the following days remains the same as in **Figure 7**. In this scenario, although the FT4 level can quickly decrease under treatment and maintain a favorable level after day 1000, the ongoing beta-cell failure cannot be halted. This indicates the early diagnosis and timely treatment of hyperthyroidism are key to mitigating diabetes.

## 4 Discussion

As a metabolic disease, diabetes has been found to be involved in crosstalk interactions with other endocrine diseases (78). Endocrine axes are the complex physiological regulatory systems connecting with each other and other body systems (e.g., the digestive system). To discover the complete glucose regulatory network, investigations of the impact of excessive hormone production on dysglycemia are necessary (1–3). Research on untangling the complex interactions of endocrine regulation would greatly facilitate the therapy for patients with secondary diabetes, in which the robust control of multiple hormone secretions is needed.

There has been an increased appreciation of the value of mathematical modeling in studying endocrine disease, which provides quantitative methods to investigate complex hormone dynamics as well as the insights to experimental research (10). Variousmathematical models have been developed focusing on different aspects of type 1 and type 2 diabetes. Nevertheless, the models exploring the influence of hormonal disorders on glycemic imbalance are sparse, retarding the advancement of precise medicine for secondary diabetes. To boost the research in secondary diabetes, we review the primary models established for the study of dysglycemia induced by excessive glucocorticoids, epinephrine, and growth hormone, respectively.

To our knowledge, there has been only one model developed to investigate the impact of excess glucocorticoids on dysglycemia. The work of Zavala et al. (25) suggests that special attention to the transient post-OGTT dynamics in patients with hypercortisolism or glucocorticoid therapy is needed to reduce the underestimate of the diabetes prevalence in hypercortisolism. The glucocorticoids-glucose-insulin model in this work is built upon the model studying the short-term glucose-insulin dynamics. A model investigating the quantitative impact of glucocorticoids on long-term glucose dynamic is also needed to facilitate a systemic understanding of chronodisruption as well as the chronotherapies for the treatment of hypercortisolism-induced diabetes. Although there are several models in the literature established to study the influence of epinephrine on the glucose regulatory system, the aim of some of the models for a further understanding of the glucose regulation under chronic stress may not be achievable, as the updated biological findings indicate that the long period effect of low-dose epinephrine can improve insulin sensitivity and reduce blood glucose level *in vivo* (31–33). Durable excessive secretion of GCs may be a more reasonable factor accounting for the dysglycemia induced by chronic stress. The effects of growth hormones on glucose regulation is intricate and changes substantially with different dosage and treatment duration (49, 51). Mathematical models studying the impact of growth hormones on the long-term glucose-insulin dynamics with varied dosage regimens are desired to help patients under durable growth hormone therapy to reduce the risk of developing dysglycemia. There has been one model established so far incorporating the influence of growth hormones to the long-term glucose regulation (52). An improvement over this model can facilitate untangling the complex interaction between growth hormones and glucose regulation, as well as the design of optimized therapy.

The quantitative impact of hyperthyroidism on diabetes progression has been studied by our hyperthyroid-diabetes model. The simulation results delineate the accumulation of excessive thyroid hormones would gradually impair glucose control, and the processcan be delayed when the progression towards hyperthyroidism is retarded. The longer time patients are exposed to hyperthyroidism, the higher risk of developing diabetes would be posed to them, even with stable thyroid hormones. The altered glucose-insulin dynamics of hyperthyroid patients after the administration of anti-thyroid drugs were analyzed upon the proposed drug-treatment model for hyperthyroidism. The results indicate that timely thyroid treatment can halt the progression of hyperglycemia and prevent beta-cell failure, underlying the reversal of diabetes. This is in line with the result from a nation-wide cohort study reporting the occurrence rate of T2DM in hyperthyroid patients decreased after thyroid treatment (69). These conclusions support the appeal that thyroid dysfunction should be managed initially in the diabetic treatment. The model may have the potential to help develop therapeutic strategies for hyperthyroidism-induced diabetes.

To reduce the complexity of the model, we incorporate merely the FT4 and TSH variables in the formulation of the hyperthyroid submodel, aiming at depicting the essential feature of the regulatory system with the fewest components. A comprehensive mathematical model of the HPT axis involving both T3-TSH interaction and deiodinase activities has been established, though the submodel per se is composed of five differential equations (79). In the hyperthyroid-diabetes model, we considered only the influence of thyroid hormones on the glucose regulation. Changes in serum TSH are also associated with the incidence of T2DM, but the connection is merely significant in hypothyroidism (57). In contrast, excessive thyroid hormones, rather than suppressed TSH level, have major effects on beta-cell apoptosis and hyperglycemia for patients with hyperthyroidism. Therefore, we neglected the impact of TSH on the glucose regulatory system in the model to avoid the introduction of additional parameters. Similarly, the influence of excessive thyroid hormones on insulin degradation was omitted for simplicity. It is of primary consideration that the assumptions underlying the model equations reflect the key characteristics of the glucose regulatory system and that the models generate plausible results in agreement with observations. aining all the concentrations of blood glucose, insulin, FT4, and TSH, we have not validated the model with individual measurements.This is a common predicament for mathematicians investigating the interaction of multiple subsystems. We envisage this obstacle would eventually be eliminated with facilitated collaborations between modelers and clinicians.

Although the model validation involves a comprehensive set of data on glucose, insulin, FT4, and TSH, which remains challenging, the model and simulations explore the possible interactions between glucose regulation and other endocrine components. isage this gap would be reduced with facilitated collaborations between modelers and clinicians. Computer simulations can be an imperative option to explore treatment strategies before the actual harm occurs to patients. Stochastic models or statistical models (80) accounting for random factors would be closer to reality than deterministic models described in this paper. However, stochastic models introduce more complexity that remains challenging to be analyzed. The deterministic models reviewed here and the new hyperthyroid-diabetes model may inspire more work with a stochastic frame in the future, further strengthening the collaboration with clinicians to enhance their applications to real cases. Although the applications of artificial intelligence (machine learning) algorithms have been become increasingly popular in manymedical domains, the formulation of such algorithms for modeling the long-term progression of diabetes remains limited (81). Time delays, which are common in modeling the metabolic system (82–85), may induce uncertainty and make machine learning challenging to capture chaotic phenomena (86, 87). No artificial intelligence work related to secondary diabetes has been established so far. By taking the initiative in secondary diabetes modeling, our work provides insights and motivations in the development of AI algorithms.

In summary, mathematical models has facilitated the understanding of the mechanism underlying the intertwined endocrine axes. Efforts on merging the glucose-insulin model with other endocrine subsystem models would promote the discovery of the entire glucose regulatory network. In this paper, we recapitulate updated biological research results for the crosstalk interactions between glucose regulatory system and other endocrine hormones. Future perspectives of mathematical modelling in the field of secondary diabetes are addressed to promote further mathematical research untangling the complexity of secondary diabetes. These efforts would facilitate the development of precise medicine forsecondary diabetes.

## Author contributions

BY wrote the original draft of the manuscript with the supervision of LR. JL, MH, and DS contributed to the methodology of the manuscript. All authors contributed to the manuscript revision, and read and approved the submitted version.

## References

[1] American Diabetes Association. Diagnosis and classification of diabetes mellitus. Diabetes Care (2010), s62–9. doi: 10.2337/dc10-S062 PMC2797383 20042775

[2] Resmini E Minuto F Colao A Ferone D. Secondary diabetes associated with principal endocrinopathies: the impact of new treatment modalities. Acta Diabetol (2009), 85–95. doi: 10.1007/s00592-009-0112-9 19322513

[3] Rouiller N Jornayvaz FR. Diabetes mellitus secondary to an endocrine pathology: when to think about it? Rev Medicale Suisse (2017), 1158–62. doi: 10.53738/REVMED.2017.13.565.1158 28639759

[4] Chwalba A Dudek A Otto-Buczkowska E. Secondary diabetes. Austin J Nutr Metab (2020), 1077.

[5] Ale A Aloro OB Adepoju A Odusan O. The spectrum of thyroid disorders at the endocrine clinic of olabisi onabanjo university teaching hospital, sagamu, south-west, Nigeria. Ann Health Res (2019), 85–92. doi: 10.30442/ahr.0501-9-39

[6] Brandt F Thvilum M Almind D Christensen K Green A Hegedüs L. Morbidity before and after the diagnosis of hyperthyroidism: a nationwide register-based study. PloS One (2013), e66711. doi: 10.1371/journal.pone.0066711 23818961 PMC3688572

[7] Wang C. The relationship between type 2 diabetes mellitus and related thyroid diseases. J Diabetes Res (2013) 2013:390534. doi: 10.1155/2013/390534 23671867 PMC3647563

[8] Sunena Mishra DN. Stress etiology of type 2 diabetes. Curr Diabetes Rev (2022), 50–6. doi: 10.2174/1573399818666220224140934 35209829

[9] Brenta G. Diabetes and thyroid disorders. Br J Diabetes Vasc Dis (2010), 172–7. doi: 10.1177/1474651410371321

[10] Zavala E Wedgwood KC Voliotis M Tabak J Spiga F Lightman SL. Mathematical modelling of endocrine systems. Trends Endocrinol Metab (2019), 244–57. doi: 10.1016/j.tem.2019.01.008 PMC6425086 30799185

[11] Ha J Sherman A. Type 2 diabetes: one disease, many pathways. Am J Physiol Endocrinol Metab (2020), E410–26. doi: 10.1152/ajpendo.00512.2019 PMC7473907 32663101

[12] Bergman RN. Origins and history of the minimal model of glucose regulation. Front Endocrinol (2021), 583016. doi: 10.3389/fendo.2020.583016 PMC7917251 33658981

[13] Alvehag K Martin C. (2006). The feedback control of glucose: on the road to type ii diabetes, in: Proceedings of the 45th IEEE Conference on Decision and Control, (San Diego, CA, USA: IEEE). pp. 685–90. Available at: https://ieeexplore.ieee.org/stamp/stamp.jsp?tp=arnumber=4177084.

[14] Vahidi O Kwok K Gopaluni R Sun L. Developing a physiological model for type ii diabetes mellitus. Biochem Eng J (2011), 7–16. doi: 10.1016/j.bej.2011.02.019

[15] De Gaetano A Hardy T Beck B Abu-Raddad E Palumbo P Bue-Valleskey J. Mathematical models of diabetes progression. Am J Physiol Endocrinol Metab (2008), E1462–79. doi: 10.1152/ajpendo.90444.2008 18780774

[16] Hardy T Abu-Raddad E Porksen N De Gaetano A. Evaluation of a mathematical model of diabetes progression against observations in the diabetes prevention program. Am J Physiol Endocrinol Metab (2012), E200–12. doi: 10.1152/ajpendo.00421.2011 22550065

[17] De Gaetano A Hardy TA. A novel fast-slow model of diabetes progression: insights into mechanisms of response to the interventions in the diabetes prevention program. PloS One (2019), e0222833. doi: 10.1371/journal.pone.0222833 31600232 PMC6786566

[18] Knowler WC Barrett-Connor E Fowler SE Hamman RF Lachin JM Walker EA. Reduction in the incidence of type 2 diabetes with lifestyle intervention or metformin. N Engl J Med (2002), 393–403. doi: 10.1056/NEJMoa012512 11832527 PMC1370926

[19] Knowler W Hamman R Edelstein S Barrett-Connor E Ehrmann D Walker E. Prevention of type 2 diabetes with troglitazone in the diabetes prevention program. Diabetes (2005), 1150–6. doi: 10.2337/diabetes.54.4.1150 PMC1351025 15793255

[20] López-Palau NE Olais-Govea JM. Mathematical model of blood glucose dynamics by emulating the pathophysiology of glucose metabolism in type 2 diabetes mellitus. Sci Rep (2020), 1–11. doi: 10.1038/s41598-020-69629-0 32728136 PMC7391357

[21] Rafacho A Ortsäter H Nadal A Quesada I. Glucocorticoid treatment and endocrine pancreas function: implications for glucose homeostasis, insulin resistance and diabetes. J Endocrinol (2014), R49–62. doi: 10.1530/JOE-14-0373 25271217

[22] Kuo T McQueen A Chen TC Wang JC. Regulation of glucose homeostasis by glucocorticoids. Glucocorticoid Signaling (2015), 99–126. doi: 10.1007/978-1-4939-2895-8_5 PMC6185996 26215992

[23] Jeong IK Oh SH Kim BJ Chung JH Min YK Lee MS. The effects of dexamethasone on insulin release and biosynthesis are dependent on the dose and duration of treatment. Diabetes Res Clin Pract (2001), 163–71. doi: 10.1016/S0168-8227(00)00229-1 11269888

[24] van Raalte DH Nofrate V Bunck MC van Iersel T Schaap JE Nässander UK. Acute and 2-week exposure to prednisolone impair different aspects of *β*-cell function in healthy men. Eur J Endocrinol (2010), 729–35. doi: 10.1530/EJE-09-1034 20124412

[25] Zavala E Gil-Gómez CA Wedgwood KC Burgess R Tsaneva-Atanasova K Herrera-Valdez MA. Dynamic modulation of glucose utilisation by glucocorticoid rhythms in health and disease. BioRxiv (2020). doi: 10.1101/2020.02.27.968354

[26] Thorens B Mueckler M. Glucose transporters in the 21st century. Am J Physiol Endocrinol Metab (2010), E141–5. doi: 10.1152/ajpendo.00712.2009 PMC2822486 20009031

[27] Palmada M Boehmer C Akel A Rajamanickam J Jeyaraj S Keller K. Sgk1 kinase upregulates glut1 activity and plasma membrane expression. Diabetes (2006), 421–7. doi: 10.2337/diabetes.55.02.06.db05-0720 16443776

[28] Unger RH. Diabetic hyperglycemia: link to impaired glucose transport in pancreatic *β*-cells. Science (1991), 1200–5. doi: 10.1126/science.2006409 2006409

[29] William Tank A Lee Wong D. Peripheral and central effects of circulating catecholamines. Compr Physiol (2011), 1–15. doi: 10.1002/cphy.c140007 25589262

[30] Hipszer BR. A mathematical model of glucose metabolism in hospitalized patients with diabetes and stress hyperglycemia. In: Drexel University dissertation (2008). Available at: https://core.ac.uk/download/pdf/190333833.pdf.

[31] Ziegler MG Elayan H Milic M Sun P Gharaibeh M. Epinephrine and the metabolic syndrome. Curr Hypertension Rep (2012), 1–7. doi: 10.1007/s11906-011-0243-6 22124970

[32] Kalinovich A Dehvari N Åslund A van Beek S Halleskog C Olsen J. Treatment with a *β*-2-adrenoceptor agonist stimulates glucose uptake in skeletal muscle and improves glucose homeostasis, insulin resistance and hepatic steatosis in mice with diet-induced obesity. Diabetologia (2020), 1603–15. doi: 10.1007/s00125-020-05171-y PMC7351816 32472192

[33] Jensen J Ruzzin J Jebens E Brennesvik E Knardahl S. Improved insulin-stimulated glucose uptake and glycogen synthase activation in rat skeletal muscles after adrenaline infusion: role of glycogen content and pkb phosphorylation. Acta Physiol Scand (2005), 121–30. doi: 10.1111/j.1365-201X.2005.01437.x 15916672

[34] Guy DA Sandoval D Richardson M Tate D Flakoll PJ Davis SN. Differing physiological effects of epinephrine in type 1 diabetes and nondiabetic humans. Am J Physiol Endocrinol Metab (2005), E178–86. doi: 10.1152/ajpendo.00310.2004 15585598

[35] Leelarathna L Little SA Walkinshaw E Tan HK Lubina-Solomon A Kumareswaran K. Restoration of self-awareness of hypoglycemia in adults with long-standing type 1 diabetes: hyperinsulinemic-hypoglycemic clamp substudy results from the hypocompass trial. Diabetes Care (2013), 4063–70. doi: 10.2337/dc13-1004 PMC3836150 24130355

[36] Mohammed II Adamu II Barka SJ. Mathematical model for the dynamics of glucose, insulin and *β*-cell mass under the effect of trauma, excitement and stress. Model Numerical Simulation Mater Sci (2019), 71–96. doi: 10.4236/mnsms.2019.94005

[37] Topp B Promislow K Devries G Miura RM T Finegood D. A model of *β*-cell mass, insulin, and glucose kinetics: pathways to diabetes. J Theor Biol (2000), 605–19. doi: 10.1006/jtbi.2000.2150 11013117

[38] Kwach B Ongati O Simwa R. Mathematical model for detecting diabetes in the blood. Appl Math Sci (2011), 279–86.

[39] Morrow LA Morganroth GS Herman WH Bergman RN Halter JB. Effects of epinephrine on insulin secretion and action in humans: interaction with aging. Diabetes (1993), 307–15. doi: 10.2337/diab.42.2.307 8425666

[40] Efendić S Luft R Cerasi E. Quantitative determination of the interaction between epinephrine and various insulin releasers in man. Diabetes (1978), 319–26. doi: 10.2337/diab.27.3.319 640237

[41] Kumar D. (2016). Modeling for diabetes detection with the help of epinephrine behavior, in: 3rd International Conference on Computing for Sustainable Global Development (INDIACom), (New Delhi, India: IEEE). pp. 1842–5. Available at: https://ieeexplore.ieee.org/stamp/stamp.jsp?tp=arnumber=7724585.

[42] Sherwin RS Shamoon H Hendler R Saccà L Eigler N Walesky M. Epinephrine and the regulation of glucose metabolism: effect of diabetes and hormonal interactions. Metabolism (1980), 1146–54. doi: 10.1016/0026-0495(80)90024-4 7001181

[43] Moscardó V Rossetti P Ampudia-Blasco FJ Bondia J. (2016). Modelling of adrenaline counterregulatory action during hypoglycaemia in type 1 diabetes, in: IEEE Conference on Control Applications (CCA), (Buenos Aires, Argentina: IEEE). pp. 430–5. Available at: https://ieeexplore.ieee.org/stamp/stamp.jsp?tp=arnumber=7587868.

[44] Bergman RN Phillips LS Cobelli C. Physiologic evaluation of factors controlling glucose tolerance in man: measurement of insulin sensitivity and beta-cell glucose sensitivity from the response to intravenous glucose. J Clin Invest (1981), 1456–67. doi: 10.1172/JCI110398 PMC370948 7033284

[45] Strobl JS Thomas MJ. Human growth hormone. Pharmacol Rev (1994), 1–34. 8190748

[46] Blethen SL Allen DB Graves D August G Moshang T Rosenfeld R. Safety of recombinant deoxyribonucleic acid-derived growth hormone: The national cooperative growth study experience. J Clin Endocrinol Metab (1996), 1704–10. doi: 10.1210/jcem.81.5.8626820 8626820

[47] Cutfield WS Wilton P Bennmarker H Albertsson-Wikland K Chatelain P Ranke MB. Incidence of diabetes mellitus and impaired glucose tolerance in children and adolescents receiving growth-hormone treatment. Lancet (2000), 610–3. doi: 10.1016/S0140-6736(99)04055-6 10696981

[48] Child CJ Zimmermann AG Scott RS Cutler GB Jr. Battelino T Blum WF. Prevalence and incidence of diabetes mellitus in GH-treated children and adolescents: analysis from the genesis observational research program. J Clin Endocrinol Metab (2011), E1025–34. doi: 10.1210/jc.2010-3023 21490076

[49] LeRoith D Yakar S. Mechanisms of disease: metabolic effects of growth hormone and insulin-like growth factor 1. Nat Clin Pract Endocrinol Metab (2007), 302–10. doi: 10.1038/ncpendmet0427 17315038

[50] Kim SH Park MJ. Effects of growth hormone on glucose metabolism and insulin resistance in human. Ann Pediatr Endocrinol Metab (2017), 145. doi: 10.6065/apem.2017.22.3.145 29025199 PMC5642081

[51] Vijayakumar A Yakar S LeRoith D. The intricate role of growth hormone in metabolism. Front Endocrinol (2011), 32. doi: 10.3389/fendo.2011.00032 PMC3356038 22654802

[52] Nora M Abdesslam B Wiam B Ali HA. A mathematical model on the effect of growth hormone on glucose homeostasis. Rev Africaine la Recherche en Informatique Mathématiques Appliquées (2019), 31–42. doi: 10.46298/arima.4945

[53] Boutayeb W Lamlili ME Boutayeb A Derouich M. (2015). The impact of obesity on predisposed people to type 2 diabetes: Mathematical model, in: International Conference on Bioinformatics and Biomedical Engineering Lecture Notes in Computer Science, (New York City: Springer). pp. 613–22. Available at: https://link.springer.com/chapter/10.1007/978-3-319-16483-059.

[54] Hall JE. Guyton and hall textbook of medical physiology e-book. Elsevier Health Sciences (2010).

[55] Yang B Tang X Haller MJ Schatz DA Rong L. A unified mathematical model of thyroid hormone regulation and implication for personalized treatment of thyroid disorders. J Theor Biol (2021), 110853. doi: 10.1016/j.jtbi.2021.110853 34358537

[56] Yang B Li J Haller MJ Schatz DA Rong L. Modeling the progression of type 2 diabetes with underlying obesity. under revision. 10.1371/journal.pcbi.1010914 PMC9997875 36848379

[57] Biondi B Kahaly GJ Robertson RP. Thyroid dysfunction and diabetes mellitus: two closely associated disorders. Endocr Rev (2019), 789–824. doi: 10.1210/er.2018-00163 30649221 PMC6507635

[58] Duntas LH Orgiazzi J Brabant G. The interface between thyroid and diabetes mellitus. Clin Endocrinol (2011), 1–9. doi: 10.1111/j.1365-2265.2011.04029.x 21521298

[59] Fukuchi M Shimabukuro M Shimajiri Y Oshiro Y Higa M Akamine H. Evidence for a deficient pancreatic *β-*cell response in a rat model of hyperthyroidism. Life Sci (2002), 1059–70. doi: 10.1016/S0024-3205(02)01791-5 12088765

[60] Dimitriadis G Raptis S. Thyroid hormone excess and glucose intolerance. Exp Clin Endocrinol Diabetes (2001), S225–39. doi: 10.1055/s-2001-18584 11460573

[61] Cavallo-Perin P Bruno A Boine L Cassader M Lenti G Pagano G. Insulin resistance in graves’ disease: a quantitative *in-vivo* evaluation. Eur J Clin Invest (1988), 607–13. doi: 10.1111/j.1365-2362.1988.tb01275.x 3147186

[62] Verga Falzacappa C Mangialardo C Madaro L Ranieri D Lupoi L Stigliano A. Thyroid hormone T3 counteracts STZ induced diabetes in mouse. PloS One (2011), e19839. doi: 10.1371/journal.pone.0019839 21637761 PMC3103518

[63] Abdalla SM Bianco AC. Defending plasma T3 is a biological priority. Clin Endocrinol (2014), 633–41. doi: 10.1111/cen.12538 PMC4699302 25040645

[64] De Castro JPW Fonseca TL Ueta CB McAninch EA Abdalla S Wittmann G. Differences in hypothalamic type 2 deiodinase ubiquitination explain localized sensitivity to thyroxine. J Clin Invest (2015), 769–81. doi: 10.1172/JCI77588 PMC4319436 25555216

[65] Larsen PR Zavacki AM. Role of the iodothyronine deiodinases in the physiology and pathophysiology of thyroid hormone action. Eur Thyroid J (2013), 232–42. doi: 10.1159/000343922 PMC3673746 23750337

[66] Lechan RM Fekete C. Role of thyroid hormone deiodination in the hypothalamus. Thyroid (2005), 883–97. doi: 10.1089/thy.2005.15.883 16131331

[67] Li H Yuan X Liu L Zhou J Li C Yang P. Clinical evaluation of various thyroid hormones on thyroid function. Int J Endocrinol (2014) 2014:e618572. doi: 10.1155/2014/618572 PMC4274666 25548564

[68] Leow MKS. A mathematical model of pituitary–thyroid interaction to provide an insight into the nature of the thyrotropin–thyroid hormone relationship. J Theor Biol (2007), 275–87. doi: 10.1016/j.jtbi.2007.05.016 17602707

[69] Chen RH Chen HY Man KM Chen SJ Chen W Liu PL. Thyroid diseases increased the risk of type 2 diabetes mellitus: A nation-wide cohort study. Medicine (2019), e15631. doi: 10.1097/MD.0000000000015631 31096476 PMC6531080

[70] Meng F Li E Yen PM Leow MKS. Hyperthyroidism in the personalized medicine era: The rise of mathematical optimization. J R Soc Interface (2019), 20190083. doi: 10.1098/rsif.2019.0083 31238837 PMC6597767

[71] Baloch Z Carayon P Conte-Devolx B Demers LM Feldt-Rasmussen U Henry JF. Laboratory medicine practice guidelines. laboratory support for the diagnosis and monitoring of thyroid disease. Thyroid: Off J Am Thyroid Assoc (2003), 3–126. doi: 10.1089/105072503321086962 12625976

[72] Znoyko SL Orlov AV Bragina VA Nikitin MP Nikitin PI. Nanomagnetic lateral flow assay for high-precision quantification of diagnostically relevant concentrations of serum TSH. Talanta (2020), 120961. doi: 10.1016/j.talanta.2020.120961 32456890

[73] American Diabetes Association. Diabetes (2021). Available at: https://www.diabetes.org/a1c/diagnosis.

[74] Ahren B Taborsky G. Beta-cell function and insulin secretion. In Ellenberg Rifkin's Diabetes Mellitus Porte D, Sherin RS, Baron A. Eds. (New York, McGraw Hill: McGraw-Hill, Health Professions Division) (2003), p. 43–65.

[75] MedicineNet. What is a high insulin level? (2021). Available at: https://www.medicinenet.com/whatisahighinsulinlevel/article.htm.

[76] SelfDecode. Fasting insulin test: Normal range + low & high levels (2021). Available at: https://labs.selfdecode.com/blog/fasting-insulin-test/.

[77] Mason CC Hanson RL Knowler WC. Progression to type 2 diabetes characterized by moderate then rapid glucose increases. Diabetes (2007), 2054–61. doi: 10.2337/db07-0053 17473220

[78] Genuth SM. Associations between diabetes and other endocrine disorders. Clin Diabetes (1990), 81–7.

[79] Berberich J Dietrich JW Hoermann R Müller MA. Mathematical modeling of the pituitary–thyroid feedback loop: role of a TSH-T3-shunt and sensitivity analysis. Front Endocrinol (2018), 91. doi: 10.3389/fendo.2018.00091 PMC5871688 29619006

[80] Keizer J. Statistical thermodynamics of nonequilibrium processes. Springer Science & Business Media (2012).

[81] Singla R Singla A Gupta Y Kalra S. Artificial intelligence/machine learning in diabetes care. Indian J Endocrinol Metab (2019), 495. doi: 10.4103/ijem.IJEM_228_19 31741913 PMC6844177

[82] Sturis J Polonsky KS Mosekilde E Van Cauter E. Computer model for mechanisms underlying ultradian oscillations of insulin and glucose. Am J Physiol Endocrinol And Metab (1991), E801–9. doi: 10.1152/ajpendo.1991.260.5.E801 2035636

[83] Song X Huang M Li J. Modeling impulsive insulin delivery in insulin pump with time delays. SIAM J Appl Mathematics (2014), 1763–85. doi: 10.1137/130933137

[84] Li J Kuang Y. Analysis of a model of the glucose-insulin regulatory system with two delays. SIAM J Appl Mathematics (2007), 757–76. doi: 10.1137/050634001

[85] Li J Kuang Y Mason CC. Modeling the glucose–insulin regulatory system and ultradian insulin secretory oscillations with two explicit time delays. J Theor Biol (2006), 722–35. doi: 10.1016/j.jtbi.2006.04.002 16712872

[86] Karamched B Hripcsak G Albers D Ott W. Delay-induced uncertainty for a paradigmatic glucose–insulin model. Chaos: Interdiscip J Nonlinear Sci (2021), 023142. doi: 10.1063/5.0027682 PMC7910007 33653035

[87] Karamched BR Hripcsak G Leibel RL Albers D Ott W. Delay-induced uncertainty in the glucose-insulin system: Pathogenicity for obesity and type-2 diabetes mellitus. Front Physiol (2022), 1786. doi: 10.3389/fphys.2022.936101 PMC9476552 36117719

